# How adverse childhood experiences impact the professional quality of life of residential care workers: resilience as a mediator for burnout, secondary traumatic stress, and compassion satisfaction

**DOI:** 10.3389/frcha.2024.1423451

**Published:** 2024-07-23

**Authors:** Lise Milne, Adrienne Ratushniak, Hannah Nguyen

**Affiliations:** ^1^ Faculty of Social Work, University of Regina, Saskatchewan, Canada; ^2^ Child Trauma Research Centre, University of Regina, Saskatchewan, Canada

**Keywords:** adverse childhood experiences, residential care workers, children and youth, resilience, trauma-informed care, compassion satisfaction, burnout, professional quality of life

## Abstract

**Introduction:**

The well-being of trauma-affected children and youth in residential care settings is contingent upon the well-being of the workers who care for them, who are increasingly expected to provide care in a trauma-informed manner. The well-being of residential care workers (RCWs) may be impacted by their own histories of adversity, their capacity individually and collectively to navigate to resources that sustain their well-being (resilience), and current perceptions of their professional quality of life.

**Objective:**

This study aimed to fill a research gap by canvassing the perspectives of RCWs to determine what and how they need to be supported in their work. We sought to better understand what personal (adverse childhood experiences, resilience) and professional (compassion satisfaction, burnout, secondary traumatic stress) experiences and capacities they bring into their work that might impact the quality of care they provide to children and youth.

**Method:**

A sample of 226 residential care workers from four residential care organizations across three Canadian provinces completed a self-report questionnaire to provide a portrait of their history of adverse childhood experiences as measured by the *ACE questionnaire*, which included two additional questions reflecting the more nuanced and expanded understanding of potential adversity in childhood in the Canadian context; their resilience, as measured by the *Adult Resilience Measure*; and compassion satisfaction, burnout, and secondary traumatic stress, as measured by the *Professional Quality of Life Measure*. Mediation was conducted to examine whether and how resilience mediated the relationship between ACEs and professional quality of life indicators.

**Results:**

(1) RCWs reported experiencing ACEs at rates much higher than general population and norm samples, especially regarding the experience of 4–5+ ACEs, known to be a threshold for increased severity in negative outcomes; (2) RCWs experienced levels of resilience and indicators of professional quality of life similar to those in other human services professions serving trauma-impacted individuals; and (3) RCW resilience significantly mediated the relationship between ACEs and compassion satisfaction, burnout, and secondary traumatic stress, and had a significant total effect for the relationship between ACEs and secondary traumatic stress. These results suggest the importance of enhancing RCW resilience in multiple ways, mainly in their professional contexts. Recommendations for resilience enhancement and suggestions for future research are provided.

## 1 Introduction

Increasing attention in research and practice are being paid to the effective preparation and support of residential care workers (RCWs), who provide direct care to youth in out-of-home group care settings (1–5). Residential care is challenging work; youth in care frequently have complex histories of adverse childhood experiences (ACEs) (6–11), which can have a myriad of short- and long-term impacts on all spheres of functioning, primarily due to the impact of severe and chronic stress on the developing brain (12–16). Mental health issues are common among youth in residential care, such as depression, anxiety, post-traumatic stress, and dissociation (7, 10, 17). Behaviours that pose a risk to themselves and/or others include aggression, substance use, sexual behaviors (10, 13, 18–20), self-harm (17), and antisocial behaviours (21). Due to these challenging presentations, RCWs often feel anger, blame, a reduced sense of self-efficacy in their work, and may exhibit over-permissiveness or overreactions in their interactions with youth (22–24), in some cases using restraints and seclusion to manage behaviour (2, 25, 26).

These reactions from RCWs can be retraumatizing for youth (22, 23) and are counter to the overarching goal of trauma-informed care (TIC). TIC seeks to prevent re-traumatization and facilitate youth and RCW resilience through education on the prevalence and impacts of trauma, and the incorporation of key principles such as safety, stability, and trusting relationships (2, 27, 28). TIC training models have proliferated in recent years—particularly in child welfare settings (1, 3, 29). An emerging trend in these models is “helping the helpers” (30, 31)—the recognition that the well-being of youth in care is contingent on the well-being of those who provide that care (32).

As part of supporting RCW well-being, it is critical to better understand personal and professional experiences that might impact their ability to understand and apply TIC principles in their interactions with youth (3, 33). For example, relevant personal experiences or characteristics can include ACEs, attachment style, and resilience characteristics. It is probable that many RCWs carry with them their own experiences of childhood adversity, although to our knowledge no studies have examined the link between ACEs and entry into residential care work. However, studies have shown higher rates of ACEs among human service workers than those in general population samples. This includes a multi-site study with a sample of direct and indirect care child welfare professionals (*N = *192) (34), and a systematic review of 17 studies with health and social care workers (*N* = 18,715) (35). In both studies, ACEs were reported at higher rates than those in general population samples from a global meta-analysis of 206 studies across 22 countries (*N *= 546,458) (36), as well as in the norm sample of the seminal *Adverse Childhood Experiences* study (*N *= 17,337) (37). Further, “direct care” child welfare professionals reported higher ACEs than indirect care child welfare professionals (34). To our knowledge, only one ACEs study included a sample of childcare staff in a residential setting (38), although the number of participants in the direct care role was not specified. The authors found higher prevalence of ACEs among their direct care child service provider sample than those in original ACE study samples (12, 39–41). Thus, to better understand potential adversity among RCWs, inquiring about ACEs and other experiences will help inform training and support programs for RCWs to optimally undertake their important and challenging work (34, 35).

Another crucial aspect of RCW capacity to understand and implement TIC can include perceptions of professional quality of life—“the quality one feels in relation to their work as a helper” [(42), p. 8], which might include peer and supervisory support (43–45), and the knowledge and attitudes they have towards TIC within their work (46, 47). Professional quality of life can impact and be impacted by work-related stressors. Literature supports higher prevalence among helping professionals of recurrent work-related stressors (31, 44, 48–55) that can contribute to burnout (gradual onset and lingering of feelings of hopelessness and fatigue that interfere with one's work performance), secondary traumatic stress (work-related secondary trauma exposure), and compassion fatigue (combination of burnout and secondary traumatic stress) (42). One major stressor is the very nature of the work with trauma-impacted children and youth (1, 34, 45, 54, 56–58). Residential care work involves significant emotional expectations. In interactions with children and youth, RCWs are frequently required to inauthentically suppress their emotions while emoting others, simultaneously maintaining strong empathetic connections (4, 56, 59). Working in a residential care facility can be emotionally and physically draining, exacerbated by disclosures of abuse, acts of aggression and violence, and other trauma responses by children or their families (52, 56, 57). It is common for affected workers to become detached or empathetically distant, consciously, or otherwise—defense mechanisms that ultimately negatively impact both themselves and the youth (31, 49, 57, 60). Often accompanying the challenge of working with trauma-impacted youth are unrealistic workloads, and/or insufficient training or administrative support (30, 47, 48, 61, 62). Helping professionals who have also had exposure to traumatic events such as ACEs are at higher risk of developing negative professional quality of life outcomes like burnout and secondary traumatic stress (1, 51, 53, 63). These can be experienced as psychological, physical, and social symptoms of these conditions, such as dysregulated emotions, brain fog, nightmares, headaches, social isolation, and anxiety (48, 52, 64).

Fortunately, resilience is thought to mediate some of the impacts of adversity in childhood (13), as well as the negative impacts of work-related stress (48, 65). A socio-ecological definition of resilience is defined as, “the capacity of individuals to navigate their way to the psychological, social, cultural, and physical resources that sustain their well-being, and their capacity individually and collectively to negotiate for these resources to be provided and experienced in culturally meaningful ways” [(66), p. 10]. Rather than emphasizing individual characteristics, current resiliency literature emphasizes a multisystemic perspective, where the focus is on the human interdependency with the socioeconomic systems in which we live (67, 68). Resilience-promoting organizational factors such as social support, healthy organizational culture, and manageable workloads, have been shown to mediate the impact of stress, as well as improve job satisfaction (45, 69). Similarly, the most protective factors have been shown within organizations that encouraged resilience, worker autonomy, and empowerment (34), with empowerment recommended specifically for direct care providers to manage the stress associated with working directly with traumatized individuals (70). Studies have also found that low resilience and unsupportive, controlling organizations were the most significant predictors for poor professional quality of life (34).

Examining resilience as a mediator may also help to understand some counter-intuitive results regarding the experience of ACEs and professional quality of life and other mental health outcomes. For example, a study involving mental health professionals found that higher ACEs were not significantly correlated with higher burnout, compassion fatigue, anxiety, or depression (71). Even more unexpected, Hiles Howard and colleagues’ (34) study found higher ACEs were in fact correlated with *lower* rates of burnout and *higher* rates of compassion satisfaction for child welfare professionals, with no significant correlation with secondary traumatic stress.

Much of the current literature on RCWs is related to TIC, including organizational implementation (2, 46), staff perceptions of TIC (33), and the impact of TIC training on the use of restraints (25). Other RCW research has focused on related areas such as primary or secondary trauma exposure and compassion fatigue (1, 52), training programs related to trauma or neurodevelopment (15), support programs that specifically support RCWs (4), the importance of workplace support, cohesion, stability (45), and the quality of youth-RCW relationships (18).

Considering gaps in research regarding deeper examinations of RCW personal and professional characteristics, the purpose of this study was to canvas RCWs to determine what and how they need to be supported. We sought to learn what experiences and capacities (i.e., ACEs and resilience) RCWs bring into their work that might impact their professional quality of life (i.e., compassion satisfaction, burnout, secondary traumatic stress), and in turn the quality of care—from a trauma-informed perspective—they can provide for trauma-impacted children and youth. We hypothesized that higher ACEs would be correlated with lower levels of resilience, compassion satisfaction, and higher levels of burnout and secondary traumatic stress. We further hypothesized that resilience would mediate the relationship between ACEs and compassion satisfaction, burnout, and secondary traumatic stress, such that higher levels of resilience would lead to improved professional quality of life outcomes.

## 2 Materials and methods

### 2.1 Research context

This exploratory study used a survey methodology to obtain demographic details and information on personal and professional experiences and characteristics of RCW participants. Four Canadian organizations across three provinces (Quebec, Manitoba, Saskatchewan) that provide residential care services to youth participated in study, which included both community-based group homes and more secure residential units for youth ages 12–17 years. Research Ethics Board (REB) approval was granted for the overall project by the researcher's institution, as well as one organization's own REB. Planning took place with participating organization liaisons to distribute the informed consent and survey online (via online platform Qualtrics) or mailed with a stamped, return addressed envelope. To protect the confidentiality of participants, no identifying information was included in the online or hardcopy surveys, and the collection of IP addresses of online survey participants was disabled in Qualtrics. Given the sensitive nature of some of the questions, participants were provided region-specific resources should they require emotional support after completing the survey.

### 2.2 Participants and procedures

Participants were 226 RCWs. Their roles are described variably across jurisdictions (e.g., Educator, Child Care Worker, Youth Care Practitioner, etc.), but their common role is to provide direct care or support to children and/or adolescents living in residential care settings, usually over 8 or 12-h shifts. Inclusion criteria were that participants be over 18 years of age, and that at the time of recruitment they had worked a minimum of 6 months in a residential care facility.

The survey included five established measures (three of which are described in this paper), which took approximately 30–45 min to complete. Demographic information was collected for the participant's organization and unit; job title and status (full-time, part-time, casual/relief); gender; ethnicity; age; highest level of education and college/university program; years in child and youth work and current position; primary duties; education and training on the impacts of trauma and, more specifically, TIC; and whether the participant felt they had received adequate education and/or training on the impacts of trauma and/or TIC. At the close of the survey, six questions were posed to elicit participants' reactions to completing the survey, using a 5-point Likert scale with response categories ranging from *strongly disagree* to *strongly agree*. As shown in Table 1, majority of participants were female and most worked full-time. The average age was 37 years. Just over 80% of participants identified as Caucasian, Indigenous, or African Canadian. The majority had a college diploma or higher, with about half educated either in child and youth care or psychology. The mean years of experience in child and youth care work was just under 11 years. Most had education (83.3%) or on-the-job training (69.0%) on the impacts of trauma, with 61.9% of staff having been educated specifically in TIC (not shown in Table). The vast majority (86.1%) felt they either did not receive adequate training on TIC (30.9%), or had, but wanted more (55.2%). Finally, median scores for participant responses to completing the questionnaire included the following (on a 5-point scale): *I found these study questions interesting* (4); *I found these study questions clear* (4), *I gained something from filling out this questionnaire* (3), *completing this questionnaire upset me more than expected* (2), *I found these study questions distressing* (2), *had I known in advance what completing this questionnaire would be like for me, I still would have agreed to participate* (4) (not shown in Table).

**Table 1:** Demographic information for residential care worker sample (*N* = 226).

	*n*	%
Gender (*N* = 226)		
Female	139	61.5
Male	79	35.0
Other/prefer not to say	8	3.5
Age (*N* = 206) (*M =* 36.9, *SD *= 12.15)		
19–30	77	37.4
31–40	58	28.1
41–50	31	15.0
51+	40	19.4
Ethnicity (*N* = 209)		
Caucasian	113	54.1
Indigenous	28	13.4
African	27	12.9
Caribbean	19	9.1
Asian	14	6.7
Other	8	4.0
Employment status (*N* = 226)		
Full-time	145	64.2
Part-time	33	14.6
Casual/relief/recall	48	21.2
Education level (*N* = 224)		
High school diploma	18	8.0
Some college/university	49	21.9
College diploma/degree	65	29.0
Bachelor's degree	77	33.4
Masters/PhD	15	6.7
Education program (*N* = 226) could select more than one		
Child and youth care	63	27.9
Psychology	51	22.6
Sociology	36	15.9
Social work	33	14.6
Other	67	25.7
Child and youth care experience (*N* = 196) (*M *=* *10.86, *SD *=* *9.74)		
<1 year	11	5.6
1–5 years	76	38.8
6–10 years	32	16.3
11–20 years	35	17.9
21 + years	42	21.4

### 2.3 Instruments

#### 2.3.1 Adverse childhood experiences

The ***Adverse Childhood Experiences* (ACE-Q) *Questionnaire*** (12) is one of the most widely used retrospective measures of childhood adversity. The measure presents examples of childhood experiences expected to negatively affect individuals, including *physical, emotional*, or *sexual abuse*; *physical* or *emotional neglect*; and *parental mental illness, substance dependence, incarceration, domestic violence, and/or separation/divorce*. To reflect the more nuanced and expanded understanding of potential adversity in childhood (72), particularly within the Canadian context, two questions were added to the original ACEs questionnaire to reflect exposure to colonial or cultural trauma (i.e., *personal* or *familial involvement in the ‘60s scoop*^1^ or *residential schools*). Thus, results for this study are presented with 12 ACEs as opposed to the original 10 ACEs, although reference is made to the general findings of the ACE-10 for comparison purposes. Response categories include *yes, no*, or *prefer not to say*. Higher scores, represented by the frequency of “*yes*” responses for the 12 questions, indicate a greater number of adverse childhood experiences. The ACE-Q was found to have adequate internal and criterion validity and acceptable internal consistency in a sample of adolescents (*α* = 0.64) (75). Per standards put forth by some authors (76), in our sample internal consistency for the ACE-Q was considered “acceptable” to “good” for both the 12-item (*α = *.79) and the 10-item (*α = *.79) questionnaires.

#### 2.3.2 Resilience

The ***Adult Resilience Measure* (RRC-ARM-2)** (77), is a 28-item measure that assesses resilience from three perspectives: *individual* (personal skills, peer support, social skills); *relational* (physical caregiving and psychological caregiving), and *contextual* (spiritual, educational, and cultural). Participants respond on a 5-point Likert-type scale (*not at all* to *a lot*). Mean subscale and total scores are calculated, with higher scores indicating a higher amount of resilience in that area. Psychometric properties are reported as “strong” (78), although some authors have suggested revisions to improving the measure (79), particularly with regards to the concept of “connectedness” (79). Measure authors report a Cronbach's alpha of .88 (77). In our sample, internal consistency for the RRC-ARM-2 was excellent (*α *= .91).

#### 2.3.3 Compassion satisfaction, burnout, secondary traumatic stress

The ***Professional Quality of Life Questionnaire* (ProQOL-V.5**) (42) is the most used measure of the positive and negative effects of helping trauma-impacted individuals (42). The 30-item instrument incorporates the effects of an individual's job/occupation into an overall assessment of how it is affecting the individual over the past 30 days. The measured positive aspect is *compassion satisfaction*, while the measured negative aspect is *compassion fatigue*, which is composed of the subscales of *burnout* and *secondary traumatic stress*. Participants rate their responses on a 5-point Likert-type scale (*never* to *very often*). Subscale scores are totaled, yielding corresponding levels (low, moderate, high). Internal reliability for both burnout and secondary traumatic stress are considered good to very strong (*α *= .84 −.90). In our sample, internal consistency was good for compassion satisfaction (*α = *.87) and secondary traumatic stress (*α *= .83), and acceptable-good for burnout (*α = *.79).

#### 2.3.4 Data analysis

Data from surveys completed online were transferred from Qualtrics to SPSS (v. 26). Data from paper surveys was entered manually into the SPSS dataset. There was very little missing data (1.3% for the ProQOL, 2.7% for the RRC-ARM-2, 5.8% for the ACE-Q), considered acceptable to conduct the analyses (80). Frequencies, measures of central tendency, and correlations were conducted.

**Figure 1:**
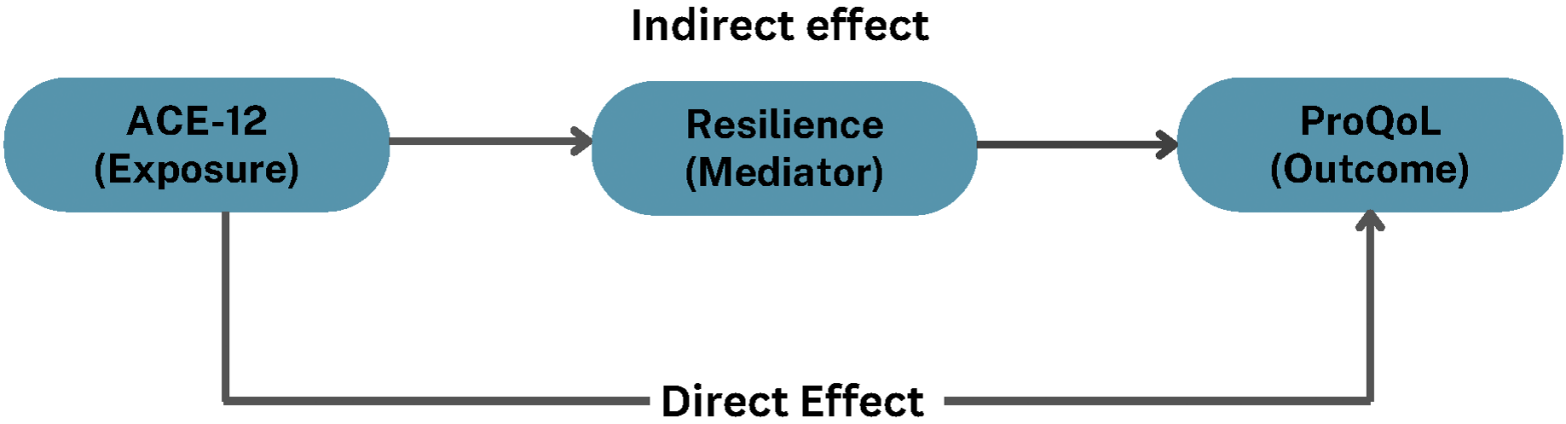
The mediation model.

As shown in Figure 1, mediation analysis was conducted to examine whether resilience (RRC-ARM-2) significantly mediated the effects of ACEs (ACE-Q) on the professional quality of life (ProQOL) indicators (burnout, secondary traumatic stress, compassion satisfaction). Mediation is a third-variable effect to explain how two variables (i.e., ACEs and burnout, ACEs and secondary traumatic stress, and ACEs and compassion satisfaction) relate and in what way. Assumptions of mediation were met through tests of linearity and normality. The Sobel Test was used to estimate the statistical significance of indirect effect in the analysis (81).

## 3 Results

### 3.1 Descriptive statistics and bivariate correlations

Table 2 presents descriptive statistics for the measures used in the study.

**Table 2:** Mean and range (minimum-maximum) for professional quality of life, adult resilience, and adverse childhood experiences measure scores.

	Mean (SD)	Min.-max.	*n*	%
Professional quality of life (ProQOL)				
Compassion satisfaction^a^ (/50)	39.26 (5.57)	24–50		
Burnout^b^ (/50)	22.24 (5.08)	10–40		
Secondary traumatic stress^c^ (/50)	22.21 (6.11)	11–38		
Adult resilience (RRC-ARM-2)^d^				
Total score (/140)	116.33 (14.18)	76–140		
Individual (/55)	47.62 (5.13)	33–55		
Relational (/35)	29.42 (5.16)	13–35		
Contextual (/50)	39.22 (6.72)	22–50		
Adverse childhood experiences				
Total ACEs (/12)	2.46 (2.63)	0–12		
0 (none)			60	26.5
1+			154	68.1
2+			112	49.6
3+			81	35.8
4+			58	25.7
5+			42	18.6

^a^ Scores of <22 for compassion satisfaction indicates a low level of compassion satisfaction (42).

^b^ Scores of <23 for burnout are good, and >41 are concerning per the ProQOL authors.

^c^ Scores of >43 for secondary traumatic stress are concerning per the ProQOL authors.

^d^ There are no norms or cut-offs provided for this measure.

#### 3.1.1 Compassion satisfaction, burnout, secondary traumatic stress

Mean and range scores fell within moderate ranges per author guidelines (i.e., between 23 and 41) (42): *compassion satisfaction* (*M* = 39.26, *SD* = 5.57), *burnout* (*M* = 22.24, *SD* = 5.08), and *secondary traumatic stress* (*M* = 22.21, *SD* = 6.11).

#### 3.1.2 Resilience

The mean total resilience score was 116.33 (*SD = *14.18) and the three subscale scores were: *individual* (*M* = 47.62, *SD* = 5.13), *relational* (*M* = 29.42, *SD* = 5.16), and *contextual* (*M* = 39.22, *SD* = 6.72).

#### 3.1.3 Adverse childhood experiences

The mean ACE-Q score for the sample was 2.46 (*SD = *2.63), with scores ranging from 0 to 12. Though just over a quarter of the sample reported no ACEs, over a quarter of participants reported 4 or more ACEs, and nearly a fifth reported 5 or more. Participants reported all ACEs included in the measure: *separation/divorce* (40.2%), *parental substance misuse* (35.5%), *parental mental illness/suicidality* (30.2%), *emotional abuse* (28.8%), *emotional neglect* (21.4%), *physical abuse* (20.5%), *parental domestic violence* (16.4%), *sexual abuse* (14.4%), *familial involvement in residential schools* (11.6%), *physical neglect* (10.7%), *parental incarceration* (9.8%), and *familial involvement in the 60s scoop* (6.1%) (not shown in Table).

Though authors of the RRC-ARM-2 and ACE-Q do not provide cut-off scores, or other interpretations of the scores, comparisons of the results with other samples are provided in the Discussion section.

### 3.2 Correlational analysis

Prior to mediation, correlational analysis using Pearson's correlation was conducted to determine the relationship among the study variables. As shown in Table 3, as hypothesized, higher ACEs were significantly negatively correlated with resilience and positively correlated with secondary traumatic stress, albeit at low (weak) levels. Also as hypothesized, resilience was positively correlated with compassion satisfaction, and negatively correlated with burnout and secondary traumatic stress, all at the *p *< .001 level, although the coefficients were also low. Though non-significant, the correlation between ACEs and compassion was contrary to our hypothesis, in that higher ACEs were correlated with higher compassion satisfaction. The findings of highly significant but weak correlations suggest that indirect pathways and/or other determinants may be impacting the variable associations.

**Table 3:** Correlation matrix of adverse childhood experiences (ACE-Q), resilience (RRC-ARM-2), compassion satisfaction, burnout, and secondary traumatic stress (ProQOL).

	ACEs	Resilience	Compassion satisfaction	Burnout	Secondary traumatic stress
ACEs	–	−.219*	.110	.028	.158*
Resilience	–	–	.401***	−.454***	−.273***
Compassion satisfaction	–	–	–	−.650***	−.305***
Burnout	–	–	–	–	.695***
Secondary traumatic stress	–	–	–	–	–

* *p *< .05; ***p < .001.

**Table 4:** The mediating effect of resilience on the relationship between adverse childhood experiences and professional quality of life indicators (compassion satisfaction, burnout, secondary traumatic stress)^a^.

	Direct Effect		Indirect effect		Total effect	
	*b*	*p*-value	*b*	*p*-value	*b*	*p*-value
ACEs on compassion satisfaction	0.439	0.001**	−0.203	0.004**	0.235	0.108
ACEs on burnout	−0.164	0.230	0.223	0.003**	0.061	0.682
ACEs on secondary traumatic stress	0.243	0.133	0.134	0.014*	0.371	0.021*

^a^ As per the adult resilience measure, the adverse childhood experiences questionnaire, and the professional quality of life questionnaire.

* *p *< .05; **p < .01.

### 3.3 Mediation analysis

**Figure 2:**
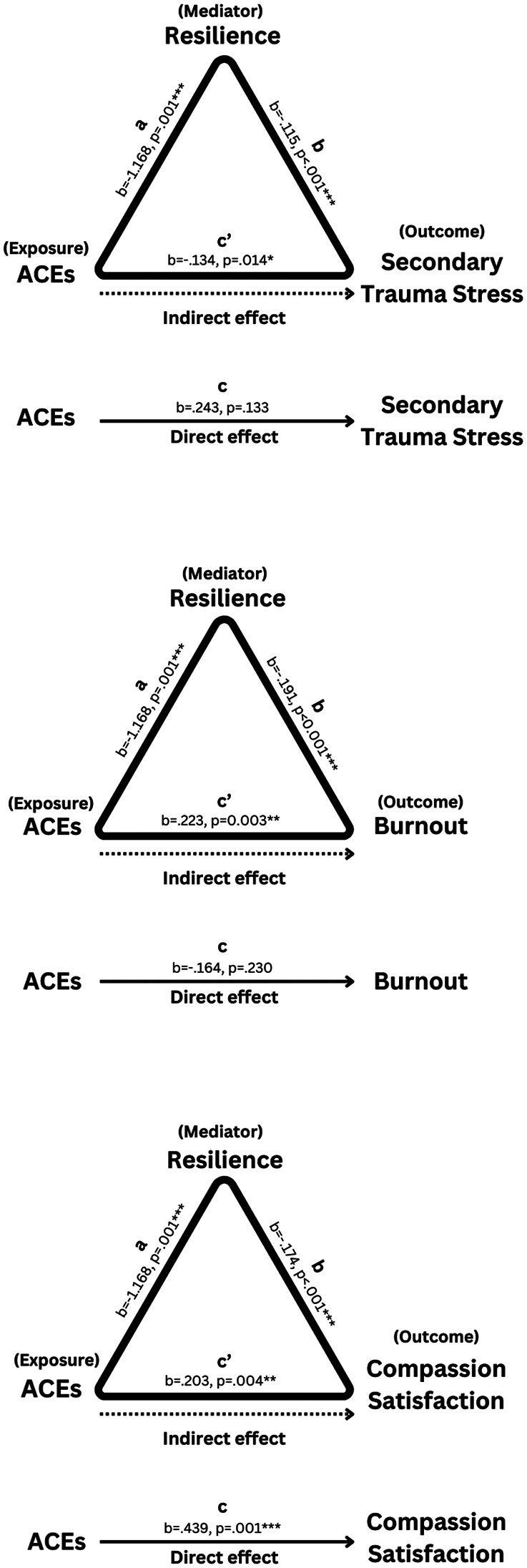
Mediation analysis—secondary traumatic stress, compassion satisfaction, burnout.

Mediation analysis revealed that resilience significantly mediated the relationship between ACEs and all three ProQOL subscales (indirect effect on secondary traumatic stress, burnout, compassion satisfaction), but there was a significant *total* effect only for the model including secondary traumatic stress (see Figure 2).

ACEs did not have a significant direct effect on **secondary traumatic stress** (i.e., higher ACEs did not directly lead to higher secondary traumatic stress), but had a significant indirect effect (*B = *0.134, *p = *.014). Resilience significantly mediated the total effects of ACEs on secondary traumatic stress (*B *= 0.371, *SE* = .159, *p* = 0.021, 95% CI = 0.057, 0.685). To further investigate the mediator, the Sobel test was utilized to examine if resilience significantly mediated the relationship between ACEs and secondary traumatic stress. The results confirmed that resilience significantly mediated the relationship (*Z* = 2.468, SE = 0.054, *p *= .014). This suggests that the total effect of ACEs on secondary traumatic stress is driven by its negative effect on resilience, which is negatively associated with secondary traumatic stress.

ACEs did not have significant total effects for **compassion satisfaction** (*B = *.0235, *SE* = 0.145, *p* = .108, 95% CI = −0.052, −0.521). However, when entering resilience as a mediator, ACEs had both a significant direct (*B* = 0.439, *SE = *0.136*, p* = .001, 95% CI = 0.170, 0.707) and indirect (*B* = −0.203, *SE* = 0.07, *p* = 0.004, 95% CI = −0.34, −0.066) effect on compassion satisfaction, such that higher ACEs resulted in lower compassion satisfaction through its negative effects on resilience, but when controlling for that negative effect, had a positive effect on compassion satisfaction (i.e., these effects balance each other out, resulting in a statistically nonsignificant total effect).

ACEs also did not have significant total effects for **burnout** (*B = *.061, *SE* = 0.148, *p* = .682, 95% CI = −0.231, −0.352). The direct effect of ACEs on burnout was nonsignificant (*B* = −.164, *SE = *0.136, *p* = .230, 95% CI = −0.433, 0.105), but the indirect effect was significant (*B *= 0.223, *SE* = 0.076, *p* = 0.003, 95% CI = 0.075, 0.371), suggesting that higher ACEs resulted in higher burnout through their shared negative associations with resilience.

Mediation analysis was also run with the original ACE-10 scores to see whether the addition of the two items would affect the results. Results were very similar, and *p*-value significance/non-significance was maintained across all results.

#### 3.3.1 Validation of the structural model

As described earlier, internal consistency was established for all three measures used in the mediation analysis. The robustness of this mediation analysis was examined for convergent and discriminant validity using Pearson's correlation coefficient (see Table 3): resilience was positively correlated with compassion satisfaction (*r *= 0.401, *p* < .001) and negatively correlated with burnout (*r* = −0.454, *p* < .001) and secondary traumatic stress (*r* = 0.273, *p* < .001), demonstrating convergent validity. ACEs were not correlated with burnout or compassion satisfaction, and showed a significant but weak correlation with secondary traumatic stress (*r *= 0.158, *p* < .05). In addition, the direct effects of ACEs on this outcome were not significant in the mediation analysis. Instead, the relationship between ACEs and all three indicators of the ProQoL was mediated by resilience through indirect effects, supporting discriminant validity by showing that ACEs do not directly or strongly impact these outcomes.

## 4 Discussion

This exploratory study aimed to fill a gap in research regarding our understanding of what personal and professional experiences and characteristics residential care workers (RCWs) bring into their work with trauma-impacted children and youth, in order to determine the conditions and supports necessary to enable them to provide trauma-informed care (TIC). The study examined whether and how RCW resilience mediates the associations between adverse childhood experiences (ACEs) and professional quality of life impacts, including compassion satisfaction, burnout, and secondary traumatic stress.

The study found that most RCWs reported at least one ACE, and over half reported at least two. Our ACE findings can be contextualized by comparing with other studies including similar professionals, as well as general population studies. Our RCW participants reported higher ACEs on the 12-item ACE-Q used in our study (*M* = 2.46, *SD* = 2.63) and the original ACE-10 (*M* = 2.26, *SD = *2.42), than a child welfare professional sample (*M* = 2.18, *SD* = 2.13) (34). For individuals reporting four or more ACEs—the threshold that experts claim lead to a significant increase in prevalence of health and social negative outcomes (12, 82)—our RCW sample and the child welfare professional sample had similar findings (25.7% vs. 25.1%). These were significantly higher than a meta-analysis general population sample (16.1%) (36) and over double that of the general population sample in the original ACEs study (12.1%) (37). Our findings also support those of other studies (34), that found higher ACEs associated with higher levels of compassion satisfaction, though they may be less counter-intuitive than they first appear. The notion of RCWs as “wounded healers” may apply. Traumatic experiences are often characterized by a lack of control (83); by entering a helping profession related to one's previous trauma, individuals can feel or perceive more control over their circumstances, “an opportunity to face these situations from a position of strength, which may be alluring to populations with ACEs” [(34), p. 446]. Also, RCWs who experienced ACEs may have had positive experiences with support from other helpers, therefore may be more likely to follow careers in helping professions (34). Given that RCWs work mainly with trauma-impacted children and youth, it is critical that wounded healers “are supported to use their own wounds to help others and not become impaired professionals whose emotional problems adversely affect their work” [(84), p. 9].

Though the authors of the RRC-ARM-2 do not include cut-off scores, our findings showed that resilience scores were similar to those found among samples of workers in similar trauma-exposed work (e.g., police), as well as samples of individuals in marginalized conditions (e.g., exposed to natural disasters, residing in crime-ridden or socio-economically poor neighbourhoods) (85).

Findings on professional quality of life indicators (compassion satisfaction, burnout, secondary traumatic stress) were not considered to be “concerning” according to the ProQOL author (42). Compared to studies with child welfare (34) and Canadian protection worker (86) samples, mean compassion satisfaction scores in our study were similar (34) or slightly higher (86), and moderately higher than the ProQOL norm sample (42). Burnout scores were lower than both child welfare and Canadian child protection worker samples, and unexpectedly on par with the ProQOL norm sample. Finally, secondary traumatic stress scores were lower than the child welfare worker sample, higher than the Canadian child protection worker sample, and significantly higher than the ProQOL norm sample. These varying findings are not unique. Indeed, some researchers have suggested that the ProQOL would benefit from revisions. For example, the inclusion of a general factor has been recommended that would reflect the continuum from compassion fatigue to compassion satisfaction, given that these concepts characterize higher and lower levels of the same construct (86). Others have suggested improvement in the coding and specific items to improve the reliability and validity of the burnout and secondary traumatic stress scales (87), confirmed in a meta-analysis of 27 studies on the factor structure of the ProQOL (88). However, the scale has shown convergent validity in its strong correlation with measures of well-being and psychological distress at work (86), and while not a diagnostic tool, can highlight important areas of well-being and concern for workers in challenging contexts.

Finally, our mediation analysis findings showed a significant indirect relationship between ACEs and all three professional quality of life indicators, as mediated by resilience. This suggests that while ACEs may not directly affect outcomes such as burnout and secondary traumatic stress, their influence is exerted through changes in resilience. Further, there was a significant total mediating effect of resilience on the relationship between ACEs and secondary traumatic stress. The absence of significant total effects for compassion satisfaction and burnout suggests that the direct impact of ACEs on professional quality of life might be masked by other contributors, such as resilience. These indirect pathways underscore the importance of finding ways to foster resilience to mitigate ACEs' negative impact on professional well-being. This indicates the need for interventions and conditions to enhance resilience, potentially further increasing compassion satisfaction, and reducing burnout and secondary traumatic stress.

The literature is replete with recommendations to support resilience enhancement of RCWs, many of which begin with how resilience can be negatively impacted by working conditions. To reflect the increasing focus on the organizational context as critical in supporting resilience enhancement for helping professionals, the recommendations here will focus mainly on this area. Frontline staff from different countries and areas of human services work overwhelmingly agree that poor working conditions are the most common contributing factors impacting retention. These conditions include unmanageable workloads, being underpaid, and most importantly, being unsupported (44, 45, 55, 89–95). Though exposure to traumatic stress has commonly been considered the most significant factor for burnout (34), chronic organizational stressors (e.g., toxic workplace cultures, poor training quality, controlling leadership, and lack of administrative support) have been identified in recent literature as the most impactful contributing factors to rates of burnout (30, 47, 51, 61, 62, 91, 96–98). Staff retention and high turnover rates are also critical contributing factors to burnout, either as reflections of burnout, or as a result of working in an environment where turnover is problematic, leading to increased work pressures that can lead to burnout. As Brend and Sprang state, “Paradoxically, this relational proximity to the children in their care also puts RCWs at risk of harmful impacts associated with secondary exposure to adverse experiences—feeding the cycle of workforce instability” [(1), p. 3]. And though burnout and turnover directly affect RCWs, high rates of turnover are a significant barrier to the successful provision of TIC for children and youth, because structure, routine, and predictability are such essential components of TIC (89). Additionally, turnover can result in challenges for children and youth to form trusting and quality relationships with RCWs, as they require safe and stable relationships due to oft-disrupted attachments (22, 99).

Creating a trauma-informed workplace culture by fostering a team environment is critical for child- and youth-serving organizations. This involves hiring people who are a good “fit” (47), and having team meetings that include many levels of workers (e.g., frontline workers, supervisors, and upper management). Open and consistent communication between frontline workers and upper management facilitates team-building, and is important for staff morale, reducing turnover, and bolstering peer support systems (4, 65, 100, 101). Creating an inclusive work culture/environment is also crucial for the safety of all workers (50), but particularly for marginalized individuals (32, 64, 94, 101). Encouraging staff empowerment by allowing more control and autonomy around their own work is an additional protective factor that promotes resiliency through reduced stress and increased compassion satisfaction (34, 53, 62, 92, 101, 102). Having leaders and management who provide consistent, supportive supervision is a major protective factor against burnout and other work-related traumatic stress for frontline workers, including RCWs (44, 65). Supportive supervision has been described as an “act of care” because it makes workers feel safer and more valued, particularly in comparison to supervision that is superficial, tokenistic, or more preoccupied with risk and surveillance (43). Reflection and mindfulness have also been identified as protective factors for worker resiliency, particularly when conducted with supervision (2, 28, 59, 101, 103, 104). Debriefing and supervised reflection is critical for facilitating safe environments for workers to process their emotions and decisions in both formal and informal contexts (102, 104, 105). Organizations should also include training for supervisors and upper management to recognize the signs of burnout and traumatic stress in their staff and learn effective ways to help them manage their stress (94).

TIC approaches to child welfare are fundamentally about relationality (22, 45, 47, 102). They aim to reduce and repair the impacts of ACEs and childhood trauma through relational security and the development of self- and interpersonal capacities. Thus, other workplace social supports, including strong informal peer support networks, have been identified as an effective method for reducing many occupational risks in child welfare (30, 44, 45, 92, 106).

A clear result from the study was that the vast majority of staff wish to have more training in TIC, even if they feel their training was “adequate”. Training can include how the brain is impacted by trauma, and its relationship to child and youth externalizing behaviors and symptoms, as well as associated therapeutic interventions (15), such as the National Child Traumatic Stress Network (NCTSN) *Trauma Training Toolkit*; the *Attachment, Self-Regulation Competency* Manual (22); and activities suggested within the *Neurosequential Model of Therapeutics* for children and youth based on enriching, therapeutic, and essential needs (107). Research has shown that such training can improve individual attitudes related to TIC (29, 33, 47, 108, 109). However, a key element of TIC is that it be situated within and across the entirety of organizations, thus RCW training alone may have little value. To address this concern, Building Resilience Through Residential Communities, a SAMHSA-funded NCTSN Treatment Services and Adaptation Center, has developed a model aimed at organizations nationwide: *Building Communities of Care* (BCC). Geared to the unique needs of residential treatment centers (RTCs), the model is considered trauma- and evidence-informed and strengths-based. BCC components include training for *all* staff; increasing access to evidence-based practices in TIC, through intensive training and technical assistance; and building “a trauma-informed workforce…through education, training and technical assistance via both intensive RTC partnerships and national dissemination activities” (p. 1) (110).

Despite such a promising model, a recent systematic review of studies on organization-wide, trauma-informed care models in out-of-home care found only seven describing three models, namely *Attachment, Self-Regulation Competency* (ARC), *Children and Residential Experiences* (CaRE), and *Sanctuary*. Though the models were seen as promising, and were shown to have significantly positive effects on children and youth in out-of-home care, they are not yet considered by the authors to be evidence-based (109). Therefore, though TIC models continue to proliferate within child well-being sectors, it is critical that research on their components (109) and perceived efficacy by RCWs, be evaluated.

Finally, though many TIC models include aspects supporting resilience enhancement of workers, we could find few interventions or programs specifically aimed at enhancing RCW resilience. A large-scale randomized control trial is currently underway in the U.S. to compare interventions for RCWs at a group home-level (*Integrated Resiliency Training and Task-sharing; IRTT*) and organization-level (virtual *Workplace Improvement Learning; WILC*) (111). However, the interventions are targeted to RCWs in congregate care settings for adults with behavioral health disabilities. Wounded Warriors Canada has developed a Trauma-Resilience Training (TRT) program, but it is specifically geared to trauma-exposed professionals such as military and public safety personnel (112).

### 4.1 Study limitations and directions for future research

While providing valuable insights into the relationships between ACEs, resilience, and professional quality of life indicators among participants, the design and mediation analysis for this study have inevitable limitations. Though the sample included data from four organizations in three provinces to provide a tri-provincial perspective, variations in provincial and organizational contexts impacts the generalizability of the findings. Further, the cross-sectional design limits the ability to infer causality among the variables (113). The findings suggest associations, but cannot establish a temporal sequence necessary to confirm a causal relationship, except in confirming the occurrence of ACEs prior to adult resilience and professional quality of life. This concern is particularly significant in mediation models, where it is crucial to establish that the mediator (resilience) temporally precedes the outcome (professional quality of life indicators). As an analysis technique, the mediation model's assumption of linear relationships between variables may not adequately represent the complex, dynamic interactions of real-world data (114). For example, unobserved confounders (e.g., an unmeasured stressor) might influence both the mediator and the outcome (115). Also, the reliance on self-reported measures may introduce recall bias and/or social desirability effects (116). Future research should utilize interventional, longitudinal (prospective and/or retrospective) designs to establish causality more effectively and assess the efficacy of specific resilience-building approaches. The inclusion of qualitative data on the personal and professional contexts of participants would enrich our understanding of the quality of life of RCWs. Similarly, information on the mental and physical health of RCWs could provide a more holistic view of the impact of ACEs and resilience on professional quality of life. Finally, expanding the sample to include a more diverse demographic profile would improve the generalizability of the findings.

## 5 Conclusion

TIC in residential care is fundamentally contingent on the well-being of RCWs. Therefore, organizations must prioritize the creation of the structural conditions necessary for RCWs to thrive (4, 30, 45, 58). This study—the first of its kind to look specifically at how for RCWs resilience mediates the impact of ACEs on compassion satisfaction, burnout, and secondary traumatic stress—demonstrates the complex interplay between personal history and professional quality of life, suggesting that supporting resilience enhancement of RCWs is crucial for improving their capacity to support vulnerable residents effectively.

## Data Availability

The raw data supporting the conclusions of this article will be made available by the authors, without undue reservation.

## Footnotes

^1^Coined by Patrick Johnston (73), the phrase “60 s scoop” (spanning the years 1960 and the mid-1980s), “describes a period in Aboriginal history in Canada in which thousands of Aboriginal children were removed from birth families and placed in non-Aboriginal environments.” [(74), p. 65].
